# Spatial Attention Is Attracted in a Sustained Fashion toward Singular Points in the Optic Flow

**DOI:** 10.1371/journal.pone.0041040

**Published:** 2012-08-08

**Authors:** Shuo Wang, Masaki Fukuchi, Christof Koch, Naotsugu Tsuchiya

**Affiliations:** 1 Computation and Neural Systems, California Institute of Technology, Pasadena, California, United States of America; 2 Division of Biology, California Institute of Technology, Pasadena, California, United States of America; 3 System and Software Platform, Sony Corporation, Shinagawa-ku, Tokyo, Japan; 4 Allen Institute for Brain Science, Seattle, Washington, United States of America; 5 Decoding and Controlling Brain Information, Japan Science and Technology Agency, Chiyoda-ku, Tokyo, Japan; 6 Brain Science Institute, RIKEN, Wako-shi, Saitama, Japan; 7 School of Psychology and Psychiatry, Monash University, Clayton, Victoria, Australia; Imperial College London, United Kingdom

## Abstract

While a single approaching object is known to attract spatial attention, it is unknown how attention is directed when the background looms towards the observer as s/he moves forward in a quasi-stationary environment. In Experiment 1, we used a cued speeded discrimination task to quantify where and how spatial attention is directed towards the target superimposed onto a cloud of moving dots. We found that when the motion was expansive, attention was attracted towards the singular point of the optic flow (the focus of expansion, FOE) in a sustained fashion. The effects were less pronounced when the motion was contractive. The more ecologically valid the motion features became (e.g., temporal expansion of each dot, spatial depth structure implied by distribution of the size of the dots), the stronger the attentional effects. Further, the attentional effects were sustained over 1000 ms. Experiment 2 quantified these attentional effects using a change detection paradigm by zooming into or out of photographs of natural scenes. Spatial attention was attracted in a sustained manner such that change detection was facilitated or delayed depending on the location of the FOE only when the motion was expansive. Our results suggest that focal attention is strongly attracted towards singular points that signal the direction of forward ego-motion.

## Introduction

The psychophysics of overt and covert attention is a well explored subject with deep roots [1]. The physiological correlates of visual attention are beginning to be understood at both the single neuron [2], [3] and at the brain regional level [4]. This has given rise to detailed computational models of the factors that control the allocation of bottom-up, saliency-driven attention in both artificial and natural static scenes [5], [6], [7].

In our daily life, however, the visual inputs to the retina are rarely stationary due to eye, head, and body movements. Furthermore, any object in the scene is embedded in a 3D environment. Looming stimuli on a 2D display are often utilized in laboratory experiments to mimic approaching objects in 3D. Looming stimuli signify biological urgencies or dangers, especially when they approach closer to the body, implying a potential interaction between motion, the projected size of an object on the retina, and attention. Therefore, to fully understand how attention works in a realistic situation, it is necessary to study how the retinal optic flow that accompanies looming stimuli, ego motion and 3D scene structures affect and guide attentional mechanisms.

Looming stimuli typically attract attention and elicit avoidance responses. Many species, including Drosophila, locusts, fiddler crabs, fishes, frogs, turtles, chicks, monkeys and humans, persistently dodge looming stimuli [8], [9], [10], [11], [12], [13], [14], [15], [16], [17], [18], [19], [20]. Infant and adult rhesus monkeys manifest persistent avoidance responses to a rapidly expanding but not to rapidly contracting circular shadows [13]. This response appears in human infants as well [8].

Indeed, the time-to-contact of an approaching object can be precisely estimated [21], [22], [23], using specialized visual mechanisms [24], [25]. Lin et al showed that a looming stimulus captures visual attention of an observer only when it would collide with him or her [26]. This effect was observed even when observers could not consciously discriminate whether or not the object was on a collision path with them [27].

While it is well known that a single looming stimulus attracts visual attention among static ones [28], little is known about whether and how visual attention is guided in the presence of an expanding optic flow where many objects loom together. Psychophysical [29], imaging [30] and physiological studies [31], [32] provided evidences that expanding optic flow can be decomposed into separate optic features and each optical feature may be individually computed and represented in the brain. Although many conventional psychophysical and electrophysiological studies of ego-motion utilized random dots for expanding optic flow [33], [34], [35], [36], [37], [38],[39],[40],[41],[42],[43],[44], such a visual stimulus is less ecological, in the sense that each individual dot does not expand in size and the distribution of the dot size is not consistent with the depth structure in the real world.

**Figure 1:**
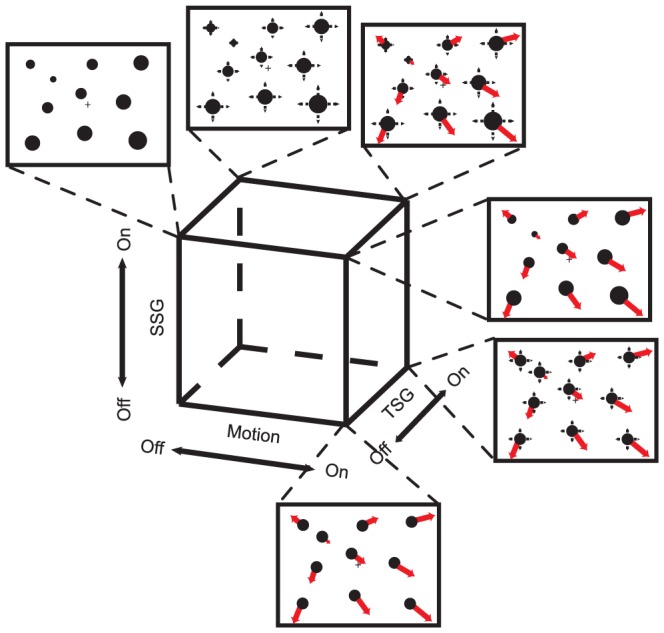
Cube representation of expanding optic flow features. Motion, the change in object size over time (or temporal size gradient, TSG), and the spatial depth structure implied by object size distribution (or spatial size gradient, SSG) correspond to one of the three axes of the cube. They can be either on or off. Each corner of the cube represents a certain combination of features. The specifications of the six conditions that went for testing are illustrated. Red arrows represent the motion. Black horizontal and vertical arrows represent the TSG. Different dot sizes represent the SSG. Note that the sizes of the dots are not to scale.

Here, we studied how attention is affected by the background visual stimuli that are composed of multiple elements. In a first experiment, we independently manipulated three features of the background dot stimuli: (1) movement of the dots away from or towards a singular point in the visual field (FOE or FOC); (2) expansion or contraction of the dots over time; (3) distribution of the size of the dots in each frame, to make it consistent or inconsistent with the depth structure of the scene in a 3D environment. We created stimuli that lacked or possessed each of the above features (see Fig. 1). We found the largest attentional effects when all three features were conjoint, emulating a situation where an observer moves toward a fronto-parallel surface in a 3D environment with depth structure.

In a second experiment, we utilized a change detection paradigm using natural scenes [45], [46], [47]. We zoomed into or out from a part of a natural scene and manipulated the location of the change in order to test if zooming motion affects spontaneous monitoring of object change. We found strong attentional effects only when the optic flow of the scene expanded (i.e., zooming towards a singular point in the scene) but not when it contracted (i.e., zooming away from the point).

## Methods

### Experiment 1: Speeded discrimination under background dot motion

#### Subjects and Apparatus

Subjects from the Caltech Community gave written informed consent. The experiments were approved by the Caltech Institutional Review Board. Fifteen subjects (6 females) and one of the authors (SW) participated in the experiments (7 subjects and SW took part in Exp 1a and the other 8 took part in Exp 1b). All subjects had good natural or corrected visual acuity.

Subjects sat 70 cm from a CRT display. The refresh rate of the display was 120 Hz and the stimuli occupied the entire display (32°×24°, visual angle). The stimuli were presented using MATLAB with the Psychtoolbox 3 [48], [49], [50] (http://psychtoolbox.org).

We monitored the subjects' eye movements with a noninvasive infrared eye-tracker (Eyelink-II system, SR Research, Canada) tracking both eyes at 250 Hz. We calibrated the eye tracker with the built-in 13-point grid method. During the main experiment, we repeated the calibration procedure when subjects had several fixation failures in a row.

#### Task

We employed a cued speeded discrimination task to quantify how attention is guided by the singular point defined by the flow field of dot motion (i.e., the focus of expansion (FOE) or contraction (FOC)) or by depth structures due to the size distribution of the dots. These features emulate some aspects of the ego-motion related optic flow and the depth structure of the 3D scene. In each trial, a singular point is randomly selected in one of the four quadrants (i.e., top-left (TL), top-right (TR), bottom-left (BL) and bottom-right (BR) corner of the screen). We define congruent, resp. incongruent, trials as those where the target was located in the same, resp. diagonally opposite, quadrant as the singular point. We define the attentional effect as the increase of the mean reaction time (RT) in the incongruent trials compared to the congruent trials.
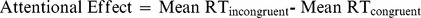
Overt eye movements are known to be attracted towards the singular point corresponding to the focus of expansion [51], [52]. To exclude a possibility that such an effect contaminates our measure of attentional effects, we monitored the gaze location and removed trials with poor fixation. We asked subjects to fixate within 1.6° from the central fixation cross and discarded trials when central fixation was broken.

**Figure 2:**
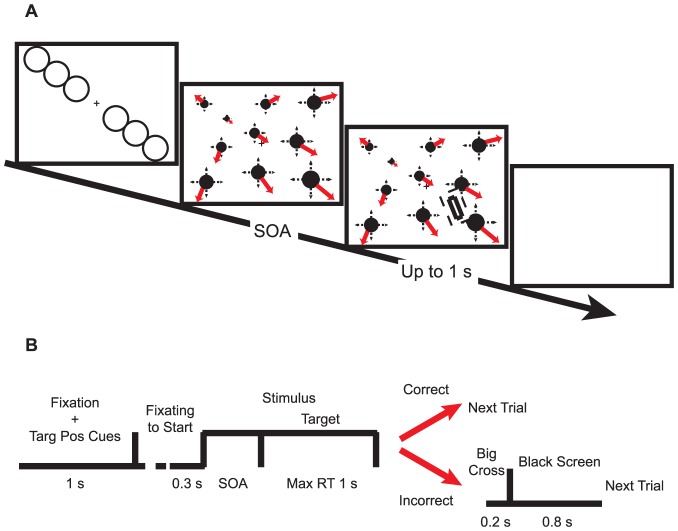
Paradigm for Experiment 1. (**A**) Structure and time course of a trial. The dashed line surrounding the tilted target rectangle demonstrates the protection zone. (**B**) A central fixation cross, together with six possible locations of the target, was shown for 1 sec. To initiate a trial, subjects had to fixate 0.3 sec stably within a 1.6 deg radius from the center of the cross. Moving dots appeared subsequently. After various SOAs, a target rectangle appeared. Subjects were asked to discriminate the tilt orientation of the target rectangle by pressing the left or right arrow key as quickly as possible. Subjects had a maximum of 1 sec to respond. If responded correctly, the next trial started. Otherwise, a big cross, indicating the error, appeared, followed by a black screen.

Figure 2 illustrates the task structure. Before each trial, a white central fixation and six thin white peripheral cueing circles (radius 2.6°) were presented for 1 sec. To test if attention is attracted exactly “to” the singular point or “towards” the side of the singular point, we measured the attentional effects at three eccentricities. The circles were positioned along the diagonal of the screen to remind the subjects of the potential locations of a target rectangle. In alternating trials, the potential locations were swapped between top-left vs. bottom-right and top-right vs. bottom-left. There were three potential locations in the top half of the screen and three in the bottom. According to their eccentricity, we refer them as ‘far’, ‘middle’ and ‘near’ cues. The singular point was always located at the ‘middle‘ eccentricity, either in the same or diagonally opposite quadrant (e.g., at the location of the top left middle or bottom right middle circle in Fig. 2). The attentional effect refers to the increase in RT between the inconsistent trials where the singular point was located in the opposite quadrant (e.g., in the top left) with respect to the target (e.g., in the bottom right, possibly near, middle or far locations) compared to the consistent trials where the singular point was located in the same quadrant as the target.

**Figure 3:**
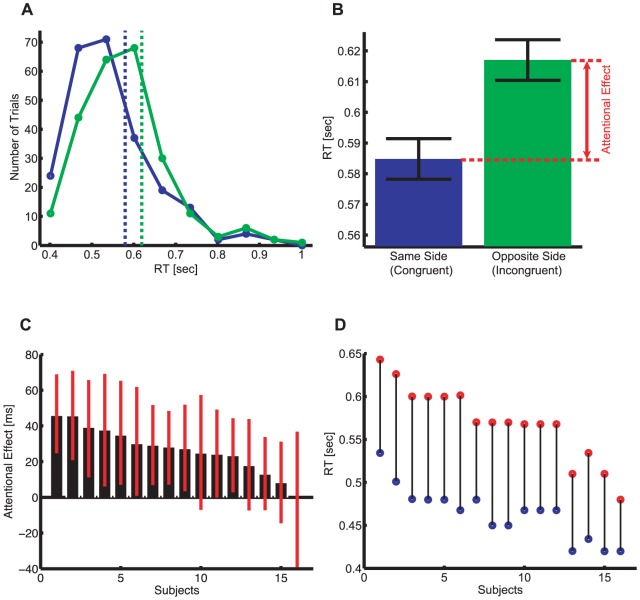
Reaction times (RTs) and the attentional effect. (**A**) RT distribution of a single subject. Blue and green colors represent congruent and incongruent trials, respectively. The dashed vertical bars represent the mean RT. (**B**) The mean RT of congruent (blue) and incongruent (green) trials of the single subject shown in (**A**). The attentional effect for each subject is defined as the increase of the mean RT in the incongruent trials compared to that in the congruent trials. Error bars denote one s.e.m. across trials. (**C**) Individual results for the attentional effect in Condition 8 (motion = on, TSG = on, SSG = on). The black bars represent the mean attentional effect and the red error bars denote the 5 to 95 percentile intervals. The means and the errors were estimated by the bootstrap method (1000 repetition per subject) [53] (**D**) Individual results of the 25th- and 75th- percentile of RT, shown in blue and red, respectively.

1 sec after the onset of fixation and cues, the subjects' eye positions were monitored. After 0.3 sec of stable fixation, a trial was initiated. The start of the trial was defined as a sudden replacement of the cues with white background dots. After a variable (0, 0.25, 0.5, 0.75, or 1 sec) and randomized stimulus onset asynchrony (SOA) with respect to the onset of the background dots, a target rectangle was presented. The target was a thick white rectangle (0.96°×3.2°), tilted either 22° left or right. The surrounding area of the target was protected from background dots by a black rectangular zone (3.2°×5.7°) tilted in the same orientation as the target to ensure its visibility (Movies S1, S2, S3, S4, S5, S6, S7, S8). To facilitate stable fixation, the central fixation cross was also protected from the background dots with a black circular exclusion zone (radius 1.6°). Subjects had to discriminate the orientation of tilt of the target (by pressing the left or right arrow key) within 1 sec from the target onset as fast as possible. When they made a mistake, the data was discarded and the trial was repeated (see below). They were told that any attribute of the background dots was task-irrelevant and independent of the location or the tilt of the target. They were asked to reduce blinks as much as possible and to keep fixation throughout the trial. Figure 3A illustrates the distribution of raw RTs and Figure 3B shows how the attentional effect is defined.

#### Stimuli

The background visual stimuli, which were irrelevant and non-informative for the discrimination task, consisted of a collection of dots. Across different conditions, we systematically manipulated three features of these dots. (1) The motion feature controlled the optic flow of the dots. In the ‘motion on’ condition, dots moved away from or towards the singular point in the display, which was located in one of the four quadrants. In the ‘motion off’ condition, the position of each dot remained the same, and did not define the location of a singular point. 2) The temporal size gradient (TSG) mimicked looming or receding of each dot. In the ‘TSG on’ condition, the radius of each dot increased or decreased over time. In the ‘TSG off’ condition, the radius of each dot remained the same. The TSG did not signify the location of the singular point. 3) The spatial size gradient (SSG) implied depth structure in the 3D environment. In the ‘SSG on’ condition, the size of the dots gradually increased proportionally to the distance from the singular point in the first frame of the stimulus movie. In the ‘SSG off’ condition, the size of the dots was uniform across the display, thus it did not signify the location of the singular point.

The SSG decides whether a scene structure is present in subsequent frames. To elucidate the relationships between the TSG and SSG, we want readers to note that the SSG decides the expanding rate of the TSG (see **Eqs. 2** and **3** and their conditions) and hence ensures whether a scene structure is present or absent across frames: If the SSG is on, the TSG makes the dots grow *proportionally* according to the distance from the singular point (the perspective of the 3D space), preserving the presence of a scene structure; otherwise, the TSG makes the dots grow *uniformly*, preserving the absence of a scene structure (see **Movies S1, S2, S3, S4, S5, S6, S7, S8**).

For all possible 8 combinations of the features, we made sample demo movies. Table 1 and its legend summarize each stimulus. To define the attentional effects, we need a singular point that is defined by the background dots. Therefore, either the ‘motion’ or the ‘SSG’ feature has to be on. Accordingly, we used 6 of the 8 conditions in our experiment. When the ‘TSG’ is turned on, it can enhance the ecological validity of the background dots. However, the TSG did not signify the location of the singular point.

**Table 1:** The stimulus parameters for each condition.

Condition (Movie)	Motion	TSG	SSG	Motion Speed	Rate of Expansion	Dot Diameter	# Trials
**1** (S1)	Off	Off	Off	0	0	0.32°	not tested
**2** (S2)	Off	Off	On	0	0	0.032°–0.64°	60
**3** (S3)	Off	On	Off	0	0.32 °/s	0.032°–0.64°	not tested
**4** (S4)	Off	On	On	0	0–1.68 °/s	0.032°–1.60°	120
**5** (S5)	On	Off	Off	0–29 °/s	0	0.32°	120
**6** (S6)	On	Off	On	0–29 °/s	0	0.032°–0.64°	120
**7** (S7)	On	On	Off	0–29 °/s	0.32 °/s	0.032°–0.64°	120
**8** (S8)	On	On	On	0–29 °/s	0–0.64 °/s	0.032°–0.64°	120

TSG and SSG stand for temporal and spatial size gradient, respectively. All conditions are exemplified in **Movies S1, S2, S3, S4, S5, S6, S7, and S8**. In Condition 1, uniformly distributed stationary dots are presented. As they do not cue the location of the singular point, this condition was not used in our experiment. In Condition 2, stationary dots with the size gradient imply a 3D scene structure, signifying the location of the singular point. In Condition 3, all the stationary dots expand their diameter at the same rate. As they do not cue the location of the singular point, this condition was not used in our experiment. In Condition 4, static dots are initially arranged with the size gradient, implying a 3D depth structure. Each dot changes its size as if it looms or recedes without changing its position. Condition 5 corresponds to a conventional random dot movie with uniform dot size, which does not change over time. In Condition 6, the initial frame has the size gradient to imply the 3D depth structure. However, each dot does not change its size as it moves, which is unlikely to happen in the real situation. In Condition 7, all the dots have the same size in the initial frame. As they start to move, they change the size together at the same rate, regardless of the distance to the singular point, which is unlikely to happen in the real situation. In Condition 8, the dots are arranged to have the size gradient to imply the 3D depth structure. Each dot changes its size as it moves so that its diameter is proportional to the distance from the singular point. This is closest to the real situation where an observer moves in a 3D environment, which has the 3D depth structure.

Note that at any given time, the speed of motion of a dot was proportional to the distance to the singular point of the flow field
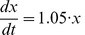
(1)in which x (in the unit of pixels) is the distance from the center of the dot to the singular point and t is in units of seconds. In the case of the contractive motion, the negative sign was added in Eq. 1. When the SSG was on (a scene structure was present), the dot size was proportional to the distance to the focus of the flow field
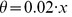
(2)in which θ is the diameter of the dot (in the unit of pixels). Thus, the rate of expansion (or contraction) of a dot was proportional to the distance to the singular point
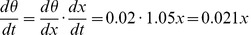
(3)When the SSG was off (no scene structure), all dots were of the same size across all frames and the rate of expansion (or contraction) of a dot was uniform regardless the distance to the focus of the flow field. The homogenous expansion (or contraction) ensures no size gradient at any frame.

In Condition 4, dots were stationary but kept on expanding in size, as if they were moving. The instantaneous rate of expansion and the dot size were proportional to the virtual distance to the singular point. This was the distance as if the dots kept on moving from their starting position. The virtual moving speed was proportional to the virtual distance to the singular point. Though no dots left the display frame, their rate of expansion increased exponentially over time. Thus, we had to terminate expansion in the middle of the trial at the frame when the largest dot reached 1.6°, in order to keep individual dots distinguishable (Movie S4). The maximum speed (here 1.6 °/s) refers to the rate of expansion of the dot that is farthest from the singular point in the last expanding frame, which had the largest expanding rate among all dots.

Conditions with the contracting motion were the reverse play of the corresponding expanding conditions. Note that, in Condition 4, since expansion stopped in the middle, its corresponding contraction started from the last expanding frame of the expansion, reverse-played all the expanding frames, and stopped and remained stationary with the first expanding frame for the rest of the time in the trial.

Five of the six tested conditions consisted of 120 trials (2 motion directions [expansion vs. contraction]×6 target locations×5 SOAs×2 sides for the singular point [top left (or top right) vs. bottom right (or bottom left)]). Condition 2 (motion off, TSG off and SSG on) was tested only for 60 trials as it did not differ between the expansion and contraction conditions (Table 1). The order of trials was fully randomized. Subjects continued until a correct trial was registered for each condition and took a break every 60 trials. In total, there were 480 or 660 correct trials (see below).

We performed two sub-experiments separately. Experiment 1a (480 trials) grouped all four conditions with motion on. We did not replace any moving dots when they moved off the screen. Over time, the background dot density decreased for expansion but increased for contraction. Experiment 1b (660 trials) grouped all six conditions, also without replacing any dots. Note that the density of dots was constant for the two conditions without motion, thus always higher than for those four conditions with motion. Everything else was the same as in Exp 1a.

#### Data Analysis

We labeled trials with poor fixation (more than 1.6° deviation from the fixation cross or a blink) or incorrect responses (incorrect target discrimination, missing response, response before 0.1 sec or after 1 sec from the target onset) as error trials, and we removed these trials from the RT analysis.

We used MATLAB for t-tests and R (R Foundation for Statistical Computing, Vienna, Austria) for repeated ANOVAs.

### Experiment 2: Change detection with zooming in and out

In Experiment 1, we tightly controlled stimuli and eye movements. Experiment 2 seeks to relax these constraints by using movies of natural scenes as stimuli and allowed eye movements in a change detection task.

#### Subjects and Apparatus

Fifteen naive male subjects, none of whom took part in Experiment 1, participated. Subjects sat 80 cm from the display with a chin rest to minimize head movements. The refresh rate of the display was 50 Hz and the images occupied the entire display (29°×22°). Eye movements were not recorded.

#### Zooming Algorithm

In Experiment 2, we chose to study the effects of attention based on natural scene images. As a consequence, we focused on Condition 8 in Experiment 1, where motion, TSG and SSG were all on. As a control, we also used Condition 1, where all three features were off. For Condition 8, singular points coincide with the FOE or FOC.

We used a zooming algorithm, based on the OpenGL function in the Psychtoolbox-3. During expansion, the camera speed of zooming was kept constant over time. The speed of expansion at each pixel was proportional to its distance (in the unit of visual angles) to the FOE and ranged from 0 to 5.4°/s. Denoting the location of a pixel p at time t during the expanding period as p(t), our zooming algorithm computes 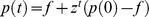 where *f* denotes the location of the FOE and *z* denotes the zoom speed, thus the p increases exponentially as t increases. The zoom speed, *z*, was fixed at 2 [°/s]. The same algorithm was used for the contraction but with negative t.

#### Procedure

Subjects pressed a button to initiate a trial. Each trial started with a 0.6 sec movie sequence consisting of 15 frames, which was replaced by a uniform gray field for 0.28 sec. The last frame of the sequence that contained a single noticeable change was then presented for 0.6 sec. After this static image, another 0.28 sec blank period followed. A complete cycle of this movie-blank-image-blank sequence was repeated until subjects pressed a space bar, indicating that they were sure that they have seen the change explicitly. When the space bar was pressed during the movie presentation or during the blank period immediately after the movie, the last frame of the movie was presented again on the screen and subjects had to indicate the change location via the mouse. When it was pressed during the stationary image or the blank period immediately following it, the stationary image was presented, on which subjects localized the change. This procedure prevented any visual transients that could be used to localize the change. If subjects could not detect the change after 52.8 sec, the trial was stopped.

#### Stimuli

For a given change detection image pair, we created 4 movie sequences for 4 different conditions corresponding to the FOE and FOC being close or far away from the location of the change. For example, when an image pair contained a change within a top-left quadrant, we created 4 movies as follows: 1. FOE-on by zooming into the top-left corner, 2. FOE-off by zooming into the bottom-right corner, 3. FOC-on by zooming out from the top-left corner, and 4. FOC-off by zooming out from the bottom-right corner. These sequences were carefully constructed such that the last frames of the 4 movies were identical. The stationary image that contained the change was also identical across all conditions. Thus, the size of the objects in the last frame of the movie and the critical change frame was identical across conditions, rendering the difficulty of the search comparable. The 5th condition, a stationary control, was created by presenting the last frame of the movie for 0.6 sec. For examples, see Movies S9, S10, S11, S12, S13.

We prepared 55 image pairs (5 of them were used for practice). We presented each image pair to a particular subject in one of five conditions. In other words, each subject was tested ten times in each condition, but each subject only saw a given image pair once in one condition. To achieve balance across subjects, we created 3 groups of 5 subjects and assigned image pairs to each group such that each image pair was seen under one experimental condition by only one member of the group. For the data analysis, the results from one group were considered as a single data point. To reflect this grouping process, the error bars are the standard deviation divided by the square root of the number of groups, which is 3.

#### Data Analysis

Prior to data collection, we defined a region of acceptable click location for each image pair by delineating a rectangular area that encompassed the change. Out of 750 trials, 701 clicks (93.5%) were within the pre-defined areas and only 10 clicks (1.3%) were outside of the rectangle. In 39 trials (5.2%), subjects did not click any location within 52.8 sec.

A one-way ANOVA was performed on log-transformed RTs because RTs were heavily long-tailed as can be seen from the cumulative histogram, whose x-axis is the logarithm of RT. For display purpose, the means of log-transformed RT as well as the error bars were transformed back into a linear scale by exponentiation. We used non-parametric Kolmogorov-Smirnov test for post-hoc comparisons.

## Results

### Experiment 1: Speeded discrimination under background dots

#### Motion is a strong cue while TSG and SSG act as auxiliary cues

Our main interest in this paper is how visual attention is attracted and guided by motion, looming stimuli, and depth structure. These cues represent some aspects of the visual input during navigation within the 3D environment. Our expansive or contractive motion as well as the size distribution of dots (the spatial size gradient, SSG) defined a singular point in the display, which may or may not attract attention. When the size of the dots changed over time (the temporal size gradient, TSG), they did not signal the location of the singular point but they assisted the ecological interpretation of the motion and depth structure of the dots. We measured whether the singular point defined by the motion and/or SSG attracted covert attention by measuring RTs in the discrimination task and by defining the attentional effect as the RT increase in the trials where the target was located in the opposite (or incongruent) side of the display from the singular point compared to where they were located in the same (or congruent) side (Fig. 3B). Significant attentional effects were highly robust and measurable in almost all subjects as shown in Figure 3C (in Condition 8), with a confidence interval estimated by the bootstrap method [53]. We demonstrated raw RT range for each subject in Figure 3D.

Comparing the overlapping conditions between Exp 1a and 1b (four conditions with motion on), we did not find any difference in the attentional effect (four-way ANOVA; Experiment [1a vs. 1b] (between-subjects factor) X motion direction [expansive vs. contractive] X TSG X SSG: the p-value for the main effect of the Experiment was >0.32). Post-hoc two-tailed t-tests confirmed no difference between each pair of overlapping conditions (all p-values were above 0.05). This analysis confirmed that our experiment was replicated by two independent samples.

**Figure 4:**
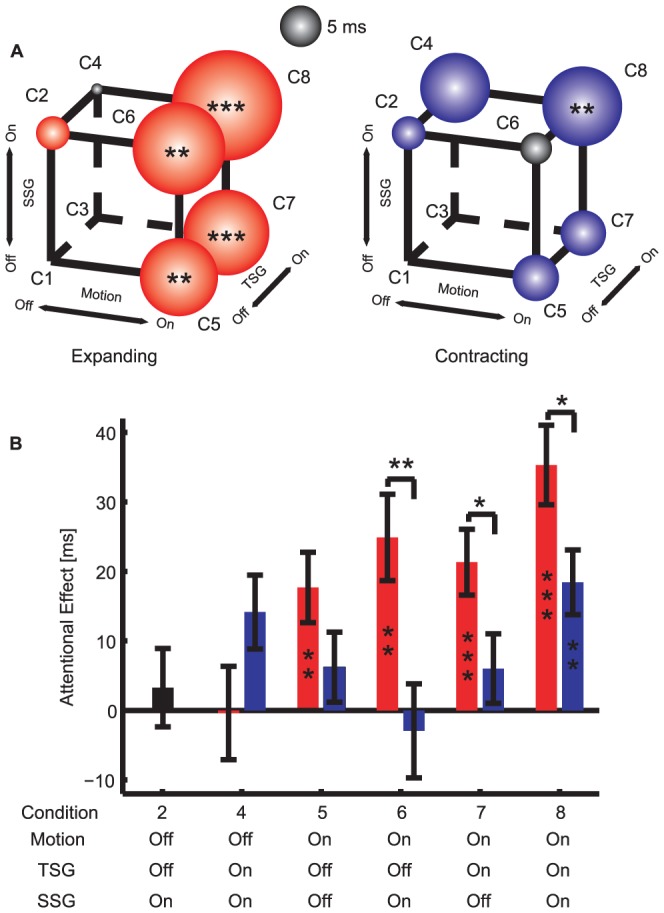
The attentional effects of the expanding and contracting optic flow. (**A**) Cube representation of the attentional effect. Red and blue colors represent the positive attentional effect for the expanding and contracting conditions, respectively. Black color represents the negative attentional effect. The area of the balls corresponds to the absolute magnitude of the attentional effect (the scale indicates 5 ms). p-values from two-tailed t-test against zero are represented by *, ** and *** indicating p<0.05, p<0.01, and p<0.001, respectively. (**B**) Bar representation of the attentional effects. Red and blue bars are for the expanding and contracting conditions, respectively. The stars indicating the level of the p-values (*, ** and ***) from two-tailed t-test against zero are shown within the bars. Significant differences between the expanding and contracting conditions are denoted by stars above the bars. Error bars denote one s.e.m. across subjects.

In Figure 4A, we represent the attentional effects as the area of balls in a cube configuration, using the motion, TSG and SSG as the three axes. Note that the condition with motion off, TSG off and SSG on (a static perspective image) was identical for expanding and contracting motion.

As the first analysis, we tested if each condition produced reliable attentional effects (two-tailed t-tests against 0). For all the conditions with the expanding motion, we observed significant attentional effects (above 0, all p<0.01, the 4 red bars on the right in Fig. 4B). Their magnitudes ranged from 18 to 35 ms for motion on, but less than 5 ms for motion off. With the contracting motion, we found significant attentional effect only when combined with the TSG and SSG [motion = on, TSG = on, SSG = on] (p<0.01, 18 ms, the rightmost blue bar in Fig. 4B). Separately for expanding and contracting motion, we compared the attentional effects between motion on and off, collapsing TSG and SSG, and found a highly significant difference for the expanding (paired t-test; p<0.0005) but not for the contracting conditions (p = 0.78). We conclude that focal attention is critically captured by the focus of expansion signaled by the expanding motion, but not by the contracting motion.

Second, we investigated the effects of the TSG with repeated ANOVAs. We used a subset of balanced data from Exp 1b, with [motion = on/off, TSG = on/off] (i.e., the data points in the upper plane of the cube in Fig. 4A). For the expanding condition, we found the main effect of motion to be significant (two-way ANOVA, p = 0.016), but neither for the main effect of TSG (p = 0.86) nor the interaction between motion and TSG (p = 0.80). For the contracting condition, we observed the main effect of TSG (p = 0.0047), but neither the main effect of motion (p = 0.95), nor the interaction between motion and TSG (p = 0.55). We conclude that the TSG was a critical feature to capture attention for the contracting but not for the expanding optic flow.

Third, we investigated the effects of the SSG on attention by confining the analysis to motion on, i.e., the right side of the cube in Figure 4A. Three-way, within-subjects repeated ANOVA (motion direction [expansion vs. contraction] X TSG X SSG) revealed significant main effects of motion direction (p<0.01) and TSG (p = 0.038) but not SSG (p = 0.09). There was a significant interaction between TSG and SSG (p = 0.044), but neither between motion direction and TSG (p>0.65), motion direction and SSG (p>0.19), nor a 3-way interaction (p>0.18). To understand the nature of the interaction between TSG and SSG, we performed post-hoc two-way ANOVAs (TSG X SSG) separately for the expanding and contracting conditions. For the expanding condition, we found a significant main effect of SSG (p = 0.021) but not TSG (p = 0.27) or interaction (p>0.41). For the contracting condition, we found a significant main effect of TSG (p = 0.043) and interaction (p = 0.026) but not the main effect of SSG (p = 0.76). Although significant, these effects tend to be small in magnitude (∼10 ms) compared to the effects caused by the presence of motion itself (∼28 ms; Fig. 4A). To conclude, the SSG played a significant role only in the expanding motion condition.

Fourth, to further characterize the importance of the TSG and SSG, we compared the attentional effects in Condition 8 (motion = on, TSG = on, SSG = on) with the conditions that lacked only the TSG and/or SSG. The effect increased with both features [motion = on, TSG = off, SSG = off] (∼18 ms, pair-wise two-tailed t-test, p = 0.026) and [motion = on, TSG = on, SSG = off] (∼14 ms, p = 0.01), but not with [motion = on, TSG = off, SSG = on] (∼10 ms, p = 0.23). This pattern seems consistent with an additive attentional effect of the TSG and SSG. To summarize, we found that the effects of the TSG and SSG were about 1/3 of the attentional effect due to the motion cue alone.

Fifth, we compared the magnitude of the attentional effects between the expanding and contracting conditions (Fig. 4B, the red and blue bars are for the expanding and contracting motion, respectively). We found that the expanding motion attracted more attention only when the TSG and/or SSG were on (paired t-test, p<0.05) but not when both the TSG and SSG were off (p = 0.12). This indicates an interdependence of motion direction, TSG and SSG. The TSG and SSG helped the expanding motion to attract attention. This further buttressed our claim that the motion plays the dominant role in the attentional effect while the TSG and SSG played an auxiliary role for the attentional attraction due to the expanding motion.

#### Target eccentricity and SOA on the attentional effects

**Figure 5:**
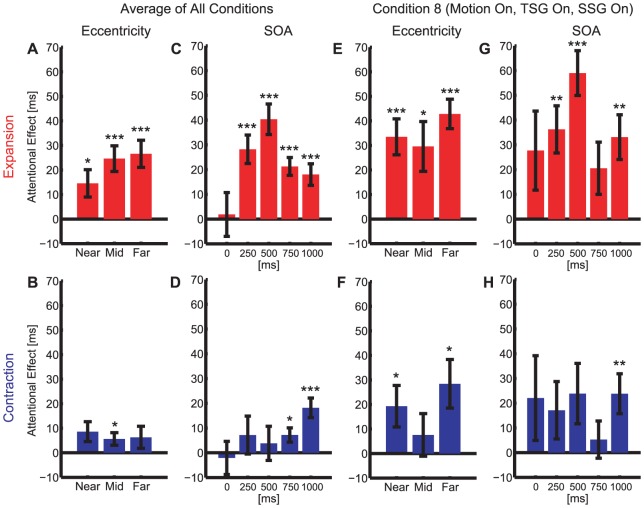
Dependency of the attentional effects on target eccentricity (A,B,E,F) and SOA (C,D,G,H) for all conditions averaged (A–D) and for Condition 8 (motion = on, SSG = on, TSG = on) (E–H). Red bars are for the expanding conditions (A,C,E,G) and blue bars are for the contracting conditions (B,D,F,H). The level of p-values from two-tailed t-test against zero are shown above the bars by *, ** and *** for p<0.05, p<0.01 and p<0.001, respectively.

So far, we showed that Condition 8 (motion = on, TSG = on, SSG = on), which is closest to the ecological condition, strongly attracts attention toward the singular point. In this section, we characterize the spatiotemporal characteristics of the attentional cueing (Fig. 5). We analyzed the influence of the target eccentricity and SOA on the attentional effects separately for the expanding (Fig. 5A, 5C, 5E and 5G) and contracting (Fig. 5B, 5D, 5F and 5H) motion, by averaging across all conditions (Fig. 5A–D) or by focusing on Condition 8 (Fig. 5E–H).

With the data averaged across all conditions (Fig. 5A–D), the results of the three-way, within-subjects ANOVA (motion direction [expansion vs. contraction] X eccentricity [near, mid and far] X SOA [0, 250, 500, 750 and 1000 ms]) revealed significant main effects of motion direction (p = 0.013) and SOA (p = 0.0022), but not eccentricity (p = 0.16). A significant interaction was observed between motion directions and SOA (p = 0.012), but no other significant interactions were observed. The lack of the main effect of the eccentricity implies that attention was attracted towards the side of the singular point and that attention was not attracted to the exact location of the singular point.

To characterize the nature of the interaction between motion direction and SOA, we performed post-hoc, one-way, within-subjects ANOVA on SOA separately for the expanding and contracting conditions (collapsing across the eccentricities): SOA dependence came from the expanding (p = 7.4×10^−5^, Fig. 5C) but not contracting conditions (p = 0.19, Fig. 5D).

The expansive motion captured attention as soon as 250 ms after stimulus onset (p<0.001 for t-tests testing that the attentional effects were above 0 at SOA = 0.25, 0.5, 0.75 and 1 s, p = 0.84 at SOA = 0 s; Fig. 5C). Unlike other exogenous attentional cues, such as a flash of a bright square, the expansive motion attracted attention rapidly and in a sustained manner (see Discussion). In contrast, the contracting motion field took a long time to capture attention (p<0.05 at SOA = 0.75 and 1 s, p>0.36 at SOA = 0, 0.25 and 0.5 s; Fig. 5D). This slow orienting process is unlikely to be caused by bottom-up stimulus factor, suggesting a possible difference in the neuronal mechanisms of attentional capture for the expansive and contractive motions.

We repeated the above analysis, focusing on Condition 8 (motion = on, TSG = on, SSG = on), which is closest to the ecological condition, as these experiments produced the largest attentional effects (Fig. 5E–H). The results were similar to those collapsing over all conditions: a marginally significant main effect of motion direction (p = 0.061) but not eccentricity (p = 0.11). Here we did not observe a significant dependency on SOA (p = 0.16). We found that the expanding motion started to attract attention for an SOA as short as 250 ms and lasting until 1 s (all p<0.05 except p = 0.071 for SOA = 0.75 s; Fig. 5G) while the contracting motion started to attract attention with the long SOA (p<0.01 at SOA = 1 s; Fig. 5H).

#### Laterality of the attentional effects

**Figure 6:**
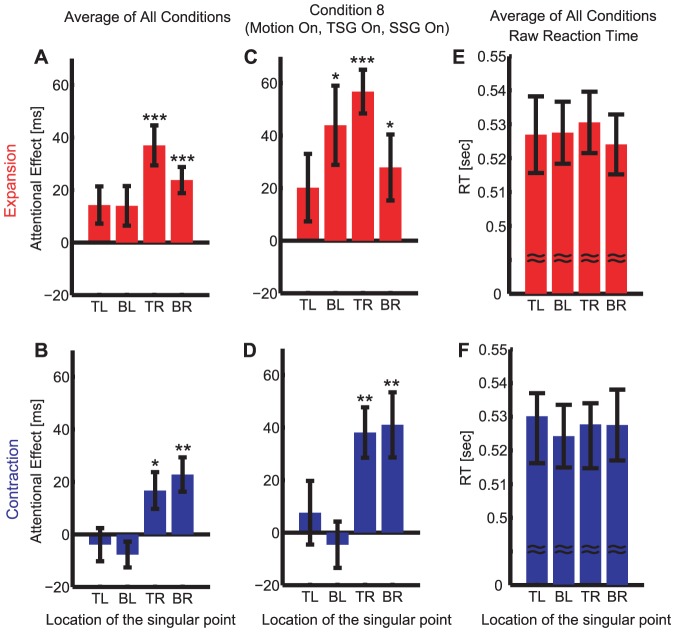
The size of the attentional effects depends on the side of the singular point. (**A, B**) The attentional effects averaged for all conditions and (**C,D**) for Condition 8 (motion on, TSG on, and SSG on). (**E,F**) The effects are not explained by the difference in raw RTs (all conditions averaged). Red bars are for the expanding conditions (**A,C,E**) and blue bars are for the contracting conditions (**B,D,F**). p-values from two-tailed t-test against zero are shown above the bars by *, ** and *** for p<0.05, p<0.01 and p<0.001, respectively. TL: top-left. TR: top-right. BL: bottom-left. BR: bottom-right.

We found an unexpected and sizable effect of laterality of the singular point. Averaging across all conditions, the attentional effects were stronger when the singular point appeared in the right visual field than the left, but they were similar between the upper and lower visual field: with a three-way within-subjects ANOVA (motion direction [expansion vs. contraction] X the horizontal [left vs. right] X the vertical [upper vs. lower] position of the singular point), we found significant main effects of motion direction (p = 0.013) and the horizontal (p = 0.021) but not the vertical position of the singular point (p = 0.41; Fig. 6A, 6B).

The most ecological motion (Condition 8, motion, TSG and SSG all on) also revealed this left-right asymmetry of the attentional effect: with a three-way within-subjects ANOVA, we found a significant main effect of the horizontal position of the singular point (p = 0.030) but not of the other factors (motion directions, p = 0.080 and the vertical position p = 0.63; Fig. 6C, 6D).

This effect is not an artifact of using the right hand for response; the reaction time for target detection was comparable when the singular point appeared in any of the quadrants (three-way within-subjects ANOVA (motion direction [expansion vs. contraction] X the horizontal X the vertical position of the singular point, the main effect of motion directions: p = 0.35; horizontal: p = 0.85; vertical p = 0.62, no significant interactions (all p>0.12; Fig. 6E, 6F).

When we grouped the trials according to the horizontal position of the target and repeated the same analysis, we did not find any significant effects.

#### Analysis of error trials

There were five types of errors (1. fixation-break, 2. wrong discrimination, 3. missing response, 4. too early response, and 5. too late response). Table 2 summarizes the error rates for each condition. All errors except fixation-break were well controlled below 5%, showing that subjects well understood and concentrated on the task. The mean rate of fixation-break was 19%. In this task, constant fixation was not easy and subjects were frequently reminded to keep a good fixation and reduce blinks. Condition 4 (motion = off, TSG = on, SSG = on) had a slightly higher error rate than the rest of conditions (two-way ANOVA (error types X conditions), the main effect of error types: p<2×10^−16^, the main effect of conditions: p = 0.017), indicating that this condition was slightly more difficult to maintain constant fixation than others. No interaction was found between the error types and conditions (p = 0.36). Separate analysis within Exp 1a and Exp 1b (two-way within-subjects ANOVA) revealed the same effect.

**Table 2:** The error rates (in percentage) for each condition.

Condition	Motion	TSG	SSG	Fixation Break	Wrong Discrimination	Missing Response	Too Early Response	Too Late Response
**1**	Off	Off	Off	-	-	-	-	-
**2**	Off	Off	On	20.6	2.17	0.116	0	0.614
**3**	Off	On	Off	-	-	-	-	-
**4**	Off	On	On	26.3	2.40	0.236	2.49	2.87
**5**	On	Off	Off	18.1	1.90	0.211	0.0324	0.541
**6**	On	Off	On	17.3	1.86	0.0403	0	0.792
**7**	On	On	Off	15.7	2.11	0.125	0	0.722
**8**	On	On	On	19.2	1.95	0.171	0	0.743
**Average**	-	-	-	19.0	2.02	0.134	0.265	0.898

### Experiment 2: Change detection with zooming in and out

**Figure 7:**
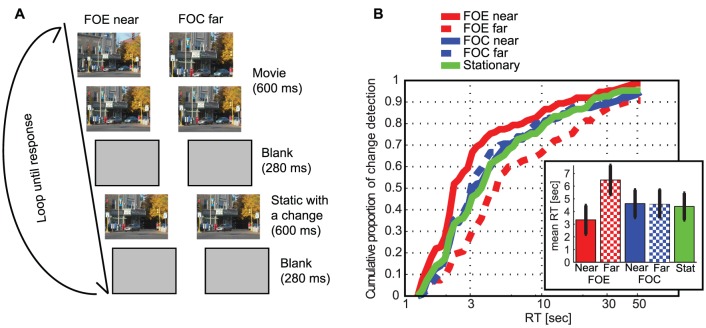
Expansion but not contraction influences the speed of change detection . (**A**) For Experiment 2, a 0.6 sec movie expanded or contracted with the associated FOE or FOC located either at the corner of the same quadrant as the change or at the opposite corner (in this example, the car on the bottom right disappeared). After a 0.28 sec blank period, a stationary image with a single noticeable change from the last frame of the movie was presented for 0.6 sec, followed by another 0.28 sec blank. This loop was repeated until subjects responded. (**B**) The cumulative detection probability as a function of RT (log scale). When the FOE was close to the location of the change, detection was facilitated, while when the FOE was far away, it interfered with change detection. Contraction did not affect change detection, compared to the control stationary condition. (Inset) Mean RT (error bars are for s.e.m.).

In Experiment 2, we investigated if the attentional effects revealed in Experiment 1 can be replicated in a more naturalistic setting. For this purpose, we used natural scene images and allowed subjects to move their eyes in a change detection paradigm (Fig. 7A).

As was expected from Experiment 1, subjects detected the change more quickly when it was close to the FOE (Fig. 7B). Mean RTs across conditions (FOE-on: 3.34±1.13 sec; FOE-off: 6.48±1.16 sec; FOC-on: 4.62±1.08 sec; FOC-off: 4.56±1.14 sec; stationary: 4.39±1.06 sec; mean±standard error) differed significantly (one-way ANOVA, p<10^−5^). A post-hoc Kolmogorov-Smirnov test confirmed (i) that the RT was strongly influenced by the location of the FOE (p<10^−9^) but not by the FOC (p>0.9), (ii) that the RT in the FOE-on condition was faster than any other conditions (p<0.01 for all comparisons) and (iii) that the RT in the FOE-off condition was slower than any other conditions (p<0.02 for all comparisons). FOC-on, FOC-off, and stationary conditions did not differ among each other (p>0.27 for all). We conclude that zooming into the change (FOE), but not zooming away from the change (FOC), guides covert and overt attention.

## Discussion

In two separate experiments, visual attention was rapidly attracted in a sustained manner towards the focus of the expanding motion. The effect was largely specific to the expanding motion and was weak or absent for the contracting motion. The motion cue played a key role in capturing attention while the temporal evolution of object size (TSG) and depth structure (SSG) played an auxiliary role (Experiment 1). Change detection was substantially slowed or facilitated depending on the location of the FOE (focus of expansion), but not FOC (focus of contraction), relative to the changed object (Experiment 2).

### Attention is attracted towards the singular point defined by the expansive, but not contractive, motion

Throughout our experiments, we found a profound asymmetry between the strong attentional effects of expansive motion and the weak or inconsistent effects for contractive motion. This ruled out a possibility that the slower speed vector fields around the singular point attracted attention since both the contractive and expansive motion had slower motion field near the singular point, yet much larger attentional effects were found in the expanding motion. Our result is consistent with the asymmetric ease in visual search (e.g., it is easy to find an expanding object among receding ones and it is difficult to find a receding object among expanding ones [54]). Likewise, cortical neurons that prefer expanding radial motion outnumber neurons that prefer contracting motion [37], [42]. The attentional and neuronal bias towards expansive motion might have been shaped through evolution reflecting ecological conditions, as contractive motion occurs only when moving backward, which happens much less often in the natural environment. This conjecture is supported by developmental studies of babies that prefer to look at expansive rather than contractive motion; even more, the developmental onset of expansive motion preference starts even before babies start moving by themselves and experiencing expansive optic flow [55], suggesting an innate bias toward expansive motion. Furthermore, in the real world, animals manifested a fine-tuned neural system to perceive expanding optic flow and control motion, for example during pigeon perching [56], fly landing [57], gannet plunge-diving [58] and during human landing from a fall [59], steering [60] and braking a car [61], [62]. Abundant psychophysical [40] and physiological [34], [63] studies have shown that these expansionary motions are processed by specialized mechanisms in mammalian visual systems.

### Sustained attentional effects

Consistent with von Muhlenen & Lleras [44] who used random dot motion, we found that the expanding optic flow field rapidly attracted attention towards the FOE in a sustained manner. While many exogenous cues attract attention, these cues tend to attract attention only during the initial several hundred milliseconds, usually acting in a repelling fashion after ∼500 ms, a phenomenon called ‘inhibition-of-return (IOR)’ [64], which is believed to facilitate orientation towards novel locations, facilitating foraging and other search behaviors.

In Experiment 1, the attentional effects were sustained up to 1 sec, which suggests that IOR is not operating for the attentional mechanisms with the expansive motion. In Experiment 2, the attentional effects even amounted to 3 sec, implying that IOR was not operating over long period of time in this paradigm. On this point, we invite readers to look at our demo movies (Movies S9, S10, S11). We expect them to feel like they tend to look at the location around the FOE repeatedly although they know that there is no change to be detected around that location. The lack of IOR in our expansive motion implies that attention towards the FOE may be important in coordinating behavior by aligning the direction of gaze, head and body.

### Mechanisms of computation of the FOE

Optic flow is processed in a network of visual motion areas, V1, V3, MT, medial superior temporal area (MST) [33], [34], [37], [42], the ventral intraparietal sulcus (VIP) [65], [66], [67], area 7a and STP (for a review, see [68]). Recordings from neurons in the ventral intraparietal sulcus (VIP), which receives strong input from MSTd, also revealed strong tuning to the optic flow [65], [66], [67]. A recent fMRI study compared the response characteristics of these two regions and found that VIP is more consistent with the computation of FOEs than MSTd [69].

Given the known strong effects of attention in VIP [2], [3] and other parietal areas, it is possible that the attentional effects of the FOE are mediated by neurons in this region. These overlapping regions for computing the FOE and attention raise the question of to which extent the FOE attracts focal attention and, if so, whether this depends on the task at hand.

### Advantage of our stimulus design

Conventional studies often used homogeneous random-dot patterns without any size change over time (TSG off) and/or uniform size distribution over space (SSG off). We found that the size change over time (TSG on) and the size distribution over space (SSG on) maximize the attentional effect of the expansive motion. Future studies might be better able to simulate ego-motion in the real world by including temporal evolvement (TSG) and depth information (SSG).

Our decomposition paradigm begs a question: how is each optical feature represented in the brain? Human psychophysical studies showed perception of visual expansion without optic flow [29], indicating that judgment of size (or scale) change is independent of local translational motion. Human fMRI studies have also tried to separate and control optical variables, such as time-to-contact, image expansion, motion in depth and rate of gap closure, in the case of looming [30]. In future research, it will be important to examine the neural mechanisms of each feature.

### Laterality effects of attention

Unexpectedly, we found the attentional effects strongly depend on the laterality of the singular point (Fig. 6A–D): when the singular point appears in the right visual field, the attentional effects became roughly twice as large (30 ms vs. 15 ms, for the expansion). Behaviorally, lateralized effects have been reported for the sensory and cognitive processing of language, face, and emotion [70]. Recent studies also report laterality effects in frogs, chickens, birds and monkeys, implying the evolutionary origin of the laterality [71]. Laterality has been also well documented for the attentional mechanisms [72]. In normal subjects, a strong asymmetry in the attentional resolution has been reported between the upper and lower visual field [73]. While it is unclear why spatial attention is more strongly captured when the singular point locates in the right visual field, our findings might be related to the ancestral origin of hemispheric lateralization for detecting unexpected predators vs. performing routine jobs [70].

### Conclusion

In this paper, we explored the attentional effects of the singular point defined by motion, object expansion and 3D depth structure. We found the strongest attentional effects in the condition that incorporates expansive motion with the 3D depth structure, which is most compatible with the visual input during forward ego motion in the 3D environment. While extensive studies have been performed on the mechanisms of attention, relatively less is explored on how attention is guided in the real 3D natural environment with the observer motion. Accordingly typical computational models of attention do not incorporate the factors we investigated here [5], [6], [7]. Our experiments revealed that expanding motion that accompanies forward ego motion is likely to guide attention strongly in everyday life. Further studies will be necessary to uncover how attention is guided and how we perceive the world in the natural environment.

## Supporting Information

Movie S1 **Demonstration of the stimulus of Experiment 1. Condition 1: [motion = off, TSG = off, SSG = off].**
(MP4)

Movie S2 **Demonstration of the stimulus of Experiment 1. Condition 2: [motion = off, TSG = off, SSG = on].**
(MP4)

Movie S3 **Demonstration of the stimulus of Experiment 1. Condition 3: [motion = off, TSG = on, SSG = off].**
(MP4)

Movie S4 **Demonstration of the stimulus of Experiment 1. Condition 4: [motion = off, TSG = on, SSG = on].**
(MP4)

Movie S5 **Demonstration of the stimulus of Experiment 1. Condition 5: [motion = on, TSG = off, SSG = off].**
(MP4)

Movie S6 **Demonstration of the stimulus of Experiment 1. Condition 6: [motion = on, TSG = off, SSG = on].**
(MP4)

Movie S7 **Demonstration of the stimulus of Experiment 1. Condition 7: [motion = on, TSG = on, SSG = off].**
(MP4)

Movie S8 **Demonstration of the stimulus of Experiment 1. Condition 8: [motion = on, TSG = on, SSG = on].**
(MP4)

Movie S9 **Demonstration of the stimulus of Experiment 2: the stationary condition.**
(MOV)

Movie S10 **Demonstration of the stimulus of Experiment 2: the condition where the FOE is far away from the change.**
(MOV)

Movie S11 **Demonstration of the stimulus of Experiment 2: the condition where the FOE is close to the change.**
(MOV)

Movie S12 **Demonstration of the stimulus of Experiment 2: the condition where the FOC is far away from the change.**
(MOV)

Movie S13 **Demonstration of the stimulus of Experiment 2: the condition where the FOC is close to the change.**
(MOV)
